# Highly efficient metallic optical incouplers for quantum well infrared photodetectors

**DOI:** 10.1038/srep30414

**Published:** 2016-07-26

**Authors:** Long Liu, Yu Chen, Zhong Huang, Wei Du, Peng Zhan, Zhenlin Wang

**Affiliations:** 1 School of Physics and National Laboratory of Solid State Microstructures, Nanjing University, Nanjing 210093, China; 2 Collaborative Innovation Center of Advanced Microstructures, Nanjing 210093, China

## Abstract

Herein, we propose a highly efficient metallic optical incoupler for a quantum well infrared photodetector (QWIP) operating in the spectrum range of 14~16 μm, which consists of an array of metal micropatches and a periodically corrugated metallic back plate sandwiching a semiconductor active layer. By exploiting the excitations of microcavity modes and hybrid spoof surface plasmons (SSPs) modes, this optical incoupler can convert infrared radiation efficiently into the quantum wells (QWs) layer of semiconductor region with large electrical field component (*E*_*z*_) normal to the plane of QWs. Our further numerical simulations for optimization indicate that by tuning microcavity mode to overlap with hybrid SSPs mode in spectrum, a coupled mode is formed, which leads to 33-fold enhanced light absorption for QWs centered at wavelength of 14.5 μm compared with isotropic absorption of QWs without any metallic microstructures, as well as a large value of coupling efficiency (*η*) of |*E*_*z*_|^2^ ~ 6. This coupled mode shows a slight dispersion over ~40° and weak polarization dependence, which is quite beneficial to the high performance infrared photodetectors.

Infrared photodetectors can convert incident infrared photons radiated from the objects to electrical signals, which could be used in a wide variety of areas, such as thermal imaging, night vision, biological spectroscopy, and sensor networking^1^. Recently, the very-long-wavelength infrared (VLWIR) photodetectors working in the wavelength regime of 14~16 μm, have been a very important topic of wide concern due to their perceived potential for applications in remote environmental monitoring, infrared earth sensing, meteorological information and astronomy research^2^,^3^. Although up to now conventional mercury cadmium telluride (MCT) is nearly the most widely used variable gap semiconductor for infrared photodetection, fabrication of MCT photodetectors with large-area focal-plane arrays is still a challenge due to the necessity of an epitaxial growth process of mercury-based compounds especially in VLWIR infrared spectral range^4^,^5^.

In recent years, quantum well infrared photodetectors (QWIPs), as an alternative choice, have developed rapidly due to their low cost, excellent reproducibility as well as high uniformity compared with the traditional MCT infrared photodetectors^6^,^7^. And the inter-subband transition energy can be varied over a wide range from short-wavelength infrared into VLWIR spectral regions by controlling the quantum well parameters^1^,^8^. However, in order to induce an inter-subband absorption, the in-coupled electric field of infrared radiation must be parallel to the growth direction of the QWs^8^. Consequently, optical in-coupling strategies in QWIPs schemes including the Brewster angle geometry^9^ and polished facet with special angle^10^ were previously utilized to obtain an electric field component normal to plane of the QWs layers, which is defined as *E*_*z*_ component. Nevertheless, the efficiency of inter-subband absorption based on these optical in-coupling methods is still fairly low^11^. In addition, regarding the applications such as infrared imaging that requires small-pixel and focal plane arrays (FPAs), it is necessary to couple infrared radiation uniformly to two-dimensional (2D) arrays of such infrared photodetection devices. To solve these issues, one-dimensional (1D) and 2D grating microstructures such as the photonic crystal slab^12^ have been proposed to serve as a grating coupler integrated on the top-surface of QWIPs to couple normally incident light into the in-plane direction, and to control the optical state in the QWs layer, which is beneficial to the inter-subband transition.

Metallic plasmonic microstructures and metamaterials are considered as ideal candidates to obtain an efficient coupling of light to a subwavelength spatial mode with a strong enhancement of localized electric field^13^,^14^,^15^,^16^,^17^. Plasmonic microstructures have been widely presented to enhance light-harvesting efficiency in order to improve photo-electron conversion for an optical-active media^18^,^19^,^20^,^21^. By introducing the coupling of localized surface plasmons (LSPs) and surface plasmon polaritons (SPPs), Chen *et al*. proposed a design of quasi-1D metallic grating to enhance the optical coupling in AlGaN/GaN QWIPs around the resonance wavelength of 4.65 μm^22^. For longer wavelength, metals resembling perfect electric conductors, highly confined resonant modes, namely SSPs, exist on the metal surface modified by periodic microstructure, which could bring the advantage of plasmonics into the longer-wavelength spectral range that is especially important for the designs of various infrared devices^23^,^24^,^25^,^26^. For example, a design principle for enhancing the infrared absorption of QWIPs has been proposed using the propagating SSPs mode introduced by a 2D gold grating^27^. Furthermore, it has been demonstrated that a perforated metal film coated with a thin high-index dielectric layer supports strongly confined hybrid SSPs due to the coupling of SSPs with the conventional guided-wave modes in the thin dielectric layer, and such a hybrid SSPs mode leads to a perfect absorption even for a thin weakly absorbing semiconductor film^28^.

On the other hand, microcavity modes existing between a metal micro-antennas and a back metal plate, provide an alternative approach to realize efficient light trapping^29^,^30^. Recently, antenna-coupled microcavities have been experimentally shown to enhance absorption of infrared light in the optical active region by tuning the microcavity mode to the inter-subband transition energy, which results in an improvement of infrared photo-detection performance^31^,^32^.

The main purposes of our metallic incoupler design are to enhance the absorption of the quantum wells layer and to overcome the limitation of non-response of QWIPs to normal incidence due to the inter-subband selection rule of quantum wells, which are the two key issues to improve the detection efficiency of QWIPs. In this letter, we propose a novel highly efficient metallic optical incoupler for enhancing the absorption of QWIPs working in the spectral regime of 14~16 μm, which comprises of a thin top-contact/QWs/bottom-contact semiconductor layer sandwiched by a periodically corrugated gold back plate and an array of gold square micropatches. This metallic microstructure could support two kinds of modes: one is the antenna-coupled microcavity mode confined in the region between the metal top-micropatch and back plate, and the other is a hybrid SSPs mode, introduced by the periodically corrugated metallic bottom plate. By optimizing the geometric parameters of this metallic microstructure, the coupling of these two modes is achieved, which leads to a large infrared absorption enhancement. Using the isotropic active region without any metallic micro-architectures as a reference, the absorption can be enhanced by 33-fold in the QWs active region using our designed microstructure. More importantly, owing to a strong mode coupling, the electric field component (*E*_*z*_) parallel to the growth direction of the QWs is much enhanced, with optical coupling coefficiency (*η*) reaching up to ~6. Meanwhile, our designed microstructure shows a broad incident angle compatible over ~40° and almost polarization independence, which might be beneficial to the infrared FPAs photodetectors.

The proposed microstructure is illustrated in Fig. 1. A mixed semiconductor layer, including QWs and top/bottom-contact GaAs layers, is sandwiched by a metallic framework composing of an 2D array of gold square micropatches with edge size marked as *a* and a bottom metal plate corrugated with a 2D grating which has the same symmetry and period (*p*) as the top micropatches array. The edge size and thickness of the bottom gold bulges are defined as *b* and *t*, respectively. In our simulations, the relative permittivity of gold is described by Drude model^33^,


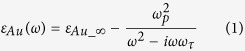


where *ω* is angular frequency of incident wave, *ε*_*Au_∞*_ is the permittivity in high frequency. We assume the value of *ε*_*Au_∞*_ can be appropriately set as 1 for the reason that it represents trasitions between the valence band and conduction band. *ω*_*p*_ = 1.37 × 10^16^ rad/s is the plasma frequency, *ω*_*τ*_ = 4.65 × 10^13^ rad/s is the damping constant of gold. The permittivity of intrinsic GaAs top- and bottom-contact layer is simulated by an oscillator model^34^,


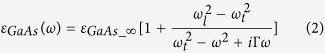


where *ω*_*l*_ = 292.1 cm^−1^ is the longitudinal-optic-phonon frequency, *ω*_*t*_ = 268.7 cm^−1^ is the transverse-optic-phonon frequency, *Γ* = 2.4 cm^−1^ is the phonon damping constant of intrinsic GaAs, and *ε*_*GaAs_∞*_ = 11.0 is the high frequency lattice dielectric constant. The QWs active region is regarded as a uniform anisotropic medium and it’s relative permittivity is taken from Ref. 25,


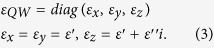


The real part *ε*′ of dielectric constant in the QWs region is the same as GaAs and the imaginary part *ε*″ of that is determined by the electronic sheet density, effective QW thickness, and oscillator strength of the inter-subband transition^35^. We chose a typical experimental value of *ε*″ = 0.5 in our simulation. As a proof-of-principle, the thickness of QWs is set as 400 nm^24^,^27^, while the thicknesses of GaAs top and bottom contact layers are fixed to 200 nm and 300 nm, respectively.

## Results

**Figure 1:**
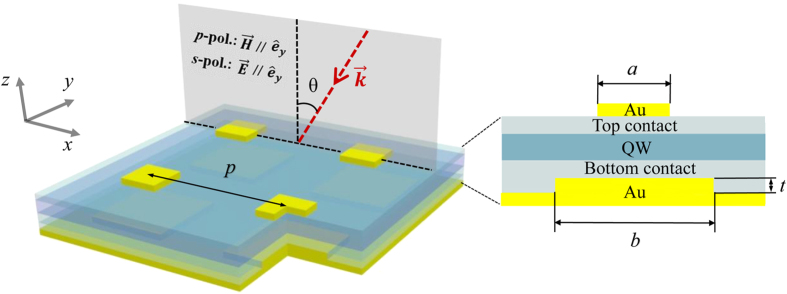
A schematic of the model device comprising of metallic microstructure and semiconductor layer. Left (3D perspective drawing): *p* is the period length, *θ* is the incident angle. Right (side-view): *a*, *b* and *t* are the edge size of the top square gold micropatches, the edge size and thickness of the corrugated gold bottom bulges, respectively. Other structural parameters are found in the main text.

As shown in Fig. 1, a microcavity is defined by the gold top-micropatch and bottom metal plate, in which a lateral Fabry-Perot resonant effect arises due to the impedance mismatch of the longitudinal cross-section along the end of top-micropatch. The periodically pattened micropatch array actually allows an efficient coupling of free space infrared photons into the optical active layer under the micropatch through the diffracted evanescent wave propagating along the surface of the micropatch array. In fact, such kind of microcavity resonances can be identified with TM_*mn*_ cavity modes confined under the gold micropatches, with the wavelength determined by the following relation:


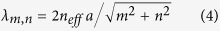


where *m* and *n* are integers counting the electric/magnetic field nodes, and *n*_*eff*_ represents the effective modal index that is related to the dispersion of semiconductor in the microcavity region^36^. In our case, for fundamental transverse-magnetic mode TM_10_ or TM_01_, *n*_*eff*_ is in the range of 4.0~4.5. It is obvious that the wavelength of microcavity resonance can be readily tuned by changing the edge size (*a*) of the top-micropatch. Alternatively, using a periodically corrugated surface of a gold bottom plate instead of a flat one, a geometry-controlled SSPs mode will be excited. The SSPs mode evanescently couples with the conventional guided-wave mode supported in the semiconductor slab with high dielectric index, and as a consequence, to form a propagating hybrid SSPs mode, leading to a higher mode confinement and higher concentration of electromagnetic energy inside the semiconductor slab^28^. The wavelength of the hybrid SSPs mode mainly depends on the period of the periodically corrugated surface, and actually the thickness of the semiconductor slab should have a certain effect on wavelength of this hybrid mode. In order to compare the infrared radiation coupling performance, the thickness of semiconductor slab including QWs and contact layers remains constant of 900 nm throughout the discussion.

**Figure 2:**
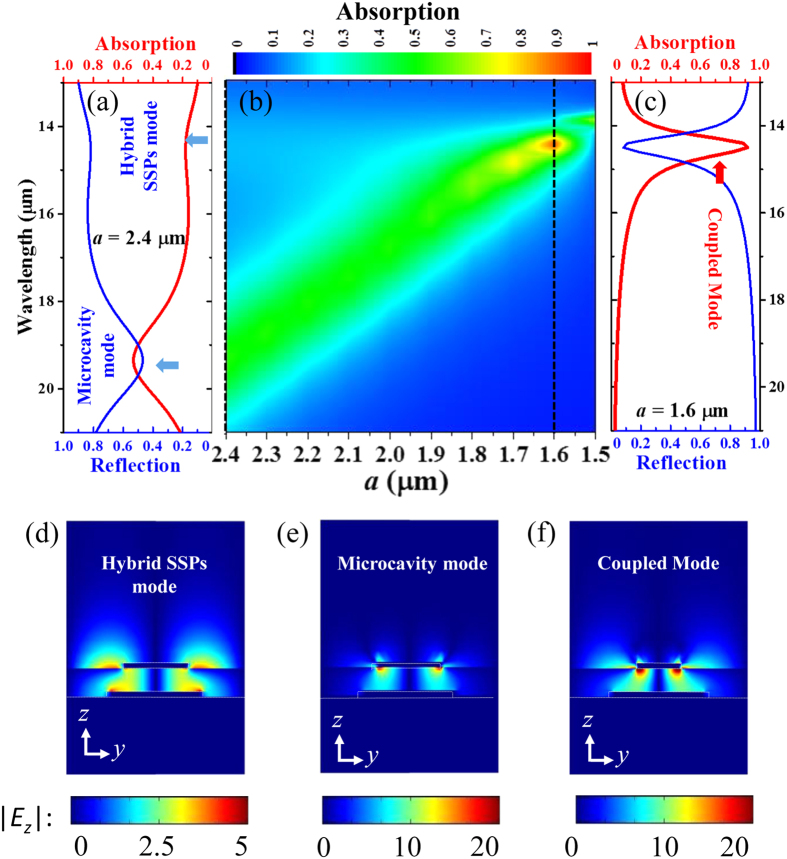
(**a,c**) are the absorption and reflection spectra with *a* = 2.4 μm and *a* = 1.6 μm, respectively. (*b*) is the absorption spectra as function of edge size (*a*) of micropatch, the black dashed lines indicate the position of *a* = 2.4 μm and *a* = 1.6 μm. (**d,e,f**) are the corresponding distributions of time averaged |*E*_*z*_| of the hybrid SSPs mode, the microcavity mode and the coupled mode in the *z*-*y* plane, respectively.

To realize the resonant absorption in the infrared regime of 14~16 μm of our interest, as a typical example, here the period (*p*), edge size (*b*) and thickness (*t*) of the gold bulges are set to *p* = 6.9 μm, *b* = 3.5 μm and *t* = 200 nm, respectively. Since the presence of an optically opaque gold back-reflector, there is no transmitted light through the sample, and thus the absorption (*A*) and reflection (*R*) satisfies the relation of *A(λ)* = 1 − *T*(*λ*) − *R*(*λ*) = 1 − *R*(*λ*). Figure 2(a) shows the absorption and reflection spectra under normal incidence of infrared radiation for a microstructure with edge size of the gold top-micropatch *a* = 2.4 μm. Two absorption bands, a weak one centered at the shorter wavelength *λ* = 14.5 μm and a strong one at the longer wavelength *λ* = 19.2 μm, are observed, which are far away from each other in the absorption spectrum. In this case, the absorption at longer wavelength is due to the excitation of the microcavity mode which is clearly shown by the contour-plots of |*E*_*z*_| illustrated in Fig. 2(e), to be a distinct lateral Fabry-Perot standing wave pattern. The absorption band located at shorter wavelength corresponds to the excitation of the propagating hybrid SSPs mode, which also could be recognized through the |*E*_*z*_| distribution in Fig. 2(d) combined with the distribution of the amplitude of the magnetic field (*H*) (result not shown here). Since the microcavity mode is quite sensitive to the top-micropatch size, it blue-shifts steadily when the gold top-micropatch size is decreasing from *a* = 2.4 μm to 1.5 μm, as is shown in Fig. 2(b). Meanwhile, because the period (*p*) of the periodically corrugated surface is fixed at 6.9 μm, the wavelength of the hybrid SSPs mode is almost unchanged.

It is worthwhile to note that a significant spectral overlap is achieved at *a* = 1.6 μm, which enables a strong coupling between the microcavity mode and the hybrid SSPs mode to form a coupled mode for which the absorption spectra are plotted in Fig. 2(c). The absorptance now reaches ~92% at wavelength of 14.5 μm, which is actually higher than the sum of 60% absorption due to the microcavity mode and 20% absorption due to the hybrid SSPs mode working separately at the 14.5 μm wavelength. The |*E*_*z*_| distribution of this coupled mode in the *z*-*y* plane is plotted in Fig. 2(f). Although, under normal incidence of infrared radiation, the incident electric field contains only *x* (or *y*) component, a strong *E*_*z*_ component is generated in the active region of QWs arising from our proposed metallic framework, which is critical to the infrared absorption in QWIPs. Comparing with that of the microcavity mode and the hybrid SSPs mode, the calculated time-averaged |*E*_*z*_| distribution of the coupled mode shows that the *E*_*z*_ distribution has a combined contribution of the two modes and a stronger electrical field enhancement.

**Figure 3:**
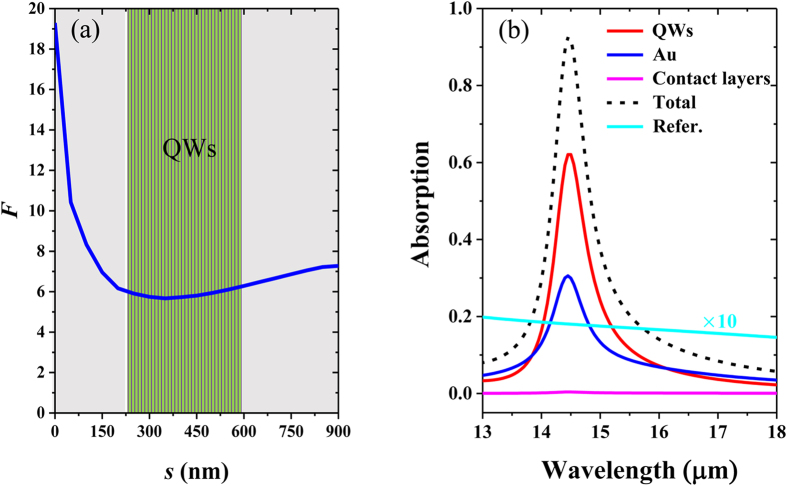
(**a**) The quantity *F* over the *x*-*y* plane as a function of the distance (*s*) away from the gold micropatch/semiconductor interface. (**b**) The absorption in different layers for the coupled mode under normal incidence of infrared radiation. The black-dashed, red, blue, and magenta lines represent the total absorption, the absorption by gold framework, the QWs layer, and both the contact layers respectively, and the cyan-dashed line shows the absorption of reference structure of the isotropic active region without any metallic framework.

To quantitatively characterize the enhancement of *E*_*z*_ component of our design at different locations in the semiconductor region comprising of the QWs layer and the top/bottom-contact layers, the quantity *F* is defined as:


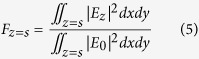


where *E*_0_ and *E*_*z*_ represent the electric field of the normal incident light and the *z* component of the induced electric field in the semiconductor layer respectively, and the integral region of above formula is the whole *x*-*y* plane located at the distance (*s*) away from the gold top-micropatch/semiconductor interface. As plotted in Fig. 3(a), *F* in the QWs region remains around 6, which means that the amplitude of |*E*_*z*_| component in the QWs active region is much higher than that of the incident electric field |*E*_0_|. The absorption of infrared incidence by the QWs layer and gold is shown in Fig. 3(b), and it is clear that quite a large proportion of infrared light (62%) is absorbed by QWs layer, which indicates that our design can efficiently coupled the incident infrared light to QWs layer. Note that the calculated absorption of gold framework is about 30%, and that of both the GaAs contact layers arising from phonon (lattice) absorption is extremely small. Furthermore, for meaningful comparison, the absorption of a reference microstructure with an isotropic active region of *ε* = *ε*′ *+* *ε*″*i* and without any metallic framework is calculated^27^, and it is observed that the absorption in the QWs active region using our proposed microstructure can be enhanced by 33 fold.

Meanwhile, taking the requirement of practical QWIPs device into account, an appropriate doping to both the GaAs contact layers should be considered^24^,^37^. The further calculation of the absorptions from different layers of QWIPs with our proposed metallic optical incoupler is performed, substituting *n*-doped GaAs contact layers with a doping concentration of *n* = 2.5 × 10^17^ cm^−3^ for the intrinsic GaAs ones. Here, the permittivity of the *n*-doped GaAs could be described as:


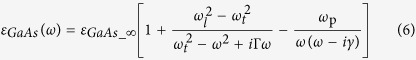


where *ω*_*l*_, *ω*_*t*_, *Γ* and *ε*_*GaAs_∞*_have the same definitions as above. *ω*_*p*_ = 168.1 cm^−1^ is the corresponding plasma frequency, and *γ* = 43.3 cm^−1^ is the scattering rate for carriers of the *n*-doped GaAs semiconductor^38^. Compared with the permittivity of intrinsic GaAs, the imaginary part of of *n*-doped GaAs as a function of wavelength will increase obviously. Thus, under this doping concentration, there exists a free carrier absorption peak from both the contact layers about 9.5% at wavelength of ~14.5 μm, and the absorption of the QWs layer slightly decreases from 62% to 59% (see Supplementary Fig. S1 for detailed simulated results), which means the coupled optical mode that we mentioned is still well confined in the region of QWs. On the other hand, the absorption band of the coupled mode presents a slight blue-shift because the real part of the permittivity of the contact layers accordingly decreases a little with the doping to intrinsic GaAs, and this slight spectral blue-shift could be easily compensated by changing a little the period of the bottom 2D grating and the edge size of the gold top-micropatch.

**Figure 4:**
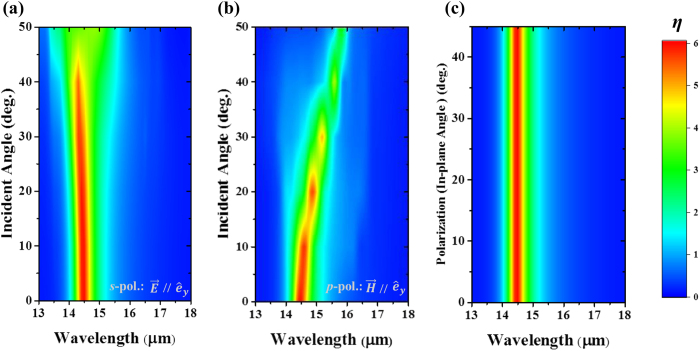
(**a,b**) the couple efficiency (*η*) of the coupled mode for *p*-, *s*-polarized incident light with incident angle from *θ* = 0° to 50°. (**c**) the couple efficiency (*η*) of the coupled mode at normal incidence for polarization angle from *ψ* = 0° to 45°.

For QWIPs devices, the photocurrent is proportional to the averaged |*E*_*z*_|^2^ across the whole QWs active region^39^. Therefore, in order to define the performance of the QWIPs based on specific architecture, commonly the couple efficiency *η* is defined as:


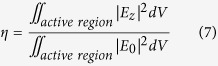


where *E*_0_ and *E*_*z*_ have been defined previously. It is generally accepted that the high-performance of a photoelectron conversion device based on light-harvesting always benefits from their large incident angle compatibility and polarization independence. For our designed metallic optical coupler for QWIPs, the couple efficiency (*η*) evolutions according to variable incident angle *θ* (from 0° to 50°) for *p*-polarized and *s*-polarized incidence are presented in Fig. 4(a,b), respectively, which show slight spectral shifts and a small intensity drop along with the increase of the incident angle, due to the non-dispersive microcavity mode^36^ and the weakly-dispersive hybrid SSPs mode^28^. As shown in Fig. 4(c), the couple efficiency of our microstructure almost shows a clear polarization independence under normal incidence because any incidence polarization can be decomposed into two orthogonal linearly polarized components and the eigen-microcavity modes TM_10_ and TM_01_ of our interest are degenerate due to the square geometry of the top-micropatch^34^. Overall, the merits of small dispersion and almost polarization independence of the metallic microstructure might be helpful to realize high-performance infrared FPAs device to detect the extremely weak infrared radiation.

**Figure 5:**
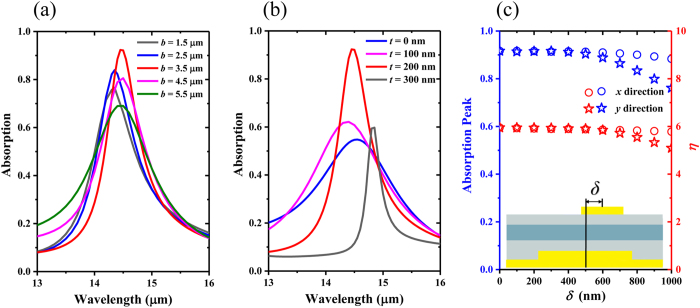
(**a**,**b**) The absorption spectrum of the hybrid structure for the coupled mode with changing the edge size (*b*) and thickness (*t*) of the corrugated gold bulges. (**c**) The intensity of absorption peak and couple efficiency with different offset (*δ*) between the geometrical center of the gold top-micropatch and that of the gold bulge along *x*-direction and *y*-direction.

Finally, the influence of the other geometric parameters on the performance of the optical incoupler, besides the size of the top-micropatch and the period of the corrugated surface that we have discussed above, are numerically investigated. Previous studies claimed that the characteristics of low-loss propagation and subwavelength field confinement for SSPs mode could be optimized through careful surface designing of the metal plate, including the groove width and depth of periodically corrugated metallic plate^25^. Changing the gold bulge’s edge size (*b*) with other parameters constant, the coupled mode presents a slight spectral shift shown in Fig. 5(a), which might be attributed to different spectral overlap between hybrid SSPs mode and the microcavity mode. In this case, the strongest absorption is achieved when *b* = 3.5 μm, and in this case the above mentioned two modes should be closest in the spectrum. On the other hand, surface fluctuation degree which could be characterized by the thickness (*t*) of the gold bulges might have some effect on coupling the infrared light to surface modes and its confinement properties. We try to display the absorption spectra of the proposed metallic optical incoupler by changing *t*, and our aim is to analyze the appropriate values for *t* that can lead to the maximum absorption caused by the *E*_*z*_ component. As shown in Fig. 5(b), spectral profiles of the absorption are fairly different according to different *t*. It is worth to note that when the *t* is 0 nm, which means the surface of the back plate is flat, the absorption peak corresponds to the excitation of microcavity mode confined in the active semiconductor layer region under the gold top-micropatch with a certain edge size of 1.6 μm. As *t* increases, allowing for the efficient excitation of the hybrid SSPs mode, the spectral positions show a clear red-shift, and a maximized absorption could be achieved with optimal *t* of ~200 nm. For a very large thickness (*t* > 300 nm), however, the excited coupled mode would be quite complicated due to the combination of hybrid SSPs mode with microcavity mode and localized modes induced by the relative high bulges^40^, and it also should be noted that in this case the pure guide-wave mode supported by the active semiconductor layer might show some differents from the case with relative low bulges, owing to the change of effective thickness of this layer (Details shown in Fig. S2 of Supplementary Information). On balance, although the wavelength of SSPs mode support by the metal back plate is determined by the period of grating and dielectric constant under the first order approximation, the grating coupling efficiency and the near field properties are actually strongly dependent on the geometric parameters of grating (e.g. the size (*b*) of the bulges and the thickness (*t*) of the gold bulges), which will eventually influence significantly the spectral properties of the hybrid SSPs mode and even those of coupled mode. In addition, the absorption response shows little dependence on the spatial offset between geometrical center of the gold top-micropatch and that of the gold bulge in the x-y plane as shown in Fig. 5(c), which indicates an excellent fault tolerance for the possible fabrication of our proposed metallic incoupler used in QWIPs in the future.

## Conslusion

In summary, a highly efficient metallic optical incoupler for a QWIPs operating in the spectrum range of 14~16 μm is proposed, which consists of an array of metal micropatches and a periodically corrugated metallic back plate that sandwich a optical-active semiconductor layer. This optical incoupler can efficiently convert free space infrared radiation into *E*_*z*_ which is the only electrical field component absorbed by QWs. By exploiting and tuning the excitation of microcavity mode and hybrid SSPs mode as well, a 33 fold enhancement of infared absorption is achieved, using a isotropic active region without any designed metallic framework as a reference. Furthermore, in this case, the value of |*E*_*z*_|^2^/|*E*_0_|^2^ reaches up to ~6, which would make a substantial contribution to improve the inter-subband transition of QWs. Note that, in this optimized metallic incoupler, the top-micropatch provides a deep-subwavelength microcavity due to its size (*a*) being much smaller than the wavelength (*λ*) of infared incidence (*a*/*λ* ≈ 10), and most of the incident electromagnetic energy could be compressed in subwavelength region where the ratio of thickness of semiconductor layer to incident wavelength is smaller than ~1/16. The coupled mode shows a small dispersion in a large incident angle range and almost polarization independence. Our novel infrared radiation incoupler shows great potential in VLWIR light harvesting with large *E*_*z*_ component, which are especially benefit to improving the performance of the QWIPs.

## Methods

Three-dimensional numerical simulations were performed using commercial software package (COMSOL Multiphysics) based on finite-element method. The periodic boundary conditions are adopted in *x* and *y* directions and a perfect matched layer condition is imposed at the boundaries along *z*-axis. Since there is a large dielectric constant difference between the air, the QWs layer, the contact layer as well as the gold layer, we use non-uniform mesh in the numerical simulations. The maximum mesh size of the god layer, the QW layer and the contact layer is 80 nm in *x-y* plane and 20 nm in *z* direction. The maximum mesh size of the air is 300 nm in *x-y* plane and 100 nm in *z* direction. The power absorbed in any material depends on the divergence of the Poynting vector and can be calculated by the relation: 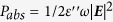 for non-magnetic materials, in which *ε*″ represents the imaginary part of the dielectric permittivity of the lossy materials and ***E*** is the total electric field. In this paper, the absorption by the quantum wells layer and the gold framework could be calculated by integrating the resistive loss over the whole region by:


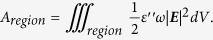


## Additional Information

**How to cite this article**: Liu, L. *et al*. Highly efficient metallic optical incouplers for quantum well infrared photodetectors. *Sci. Rep.* **6**, 30414; doi: 10.1038/srep30414 (2016).

## Supplementary Material

Supplementary Information
