# Supplementary material for: Highly efficient metallic optical incouplers for quantum well infrared photodetectors
Source: Sci Rep. 2016 Jul 26;6:30414. doi: 10.1038/srep30414 (PMC4960657; doi:10.1038/srep30414)
Supplement: Supplementary Information [file srep30414-s1.pdf]

Supplementary Information for

Highly efficient metallic optical incouplers for quantum well infrared photodetectors

Long Liu,[1] Yu Chen,[1] Zhong Huang,[1] Wei Du,[1] Peng Zhan,[1,2,*] and Zhenlin Wang[1,2,*]

[1]School of Physics and National Laboratory of Solid State Microstructures, Nanjing University, Nanjing 210093, China

[2] Collaborative Innovation Center of Advanced Microstructures, Nanjing 210093, China

*e-mail: zhanpeng@nju.edu.cn, zlwang@nju.edu.cn

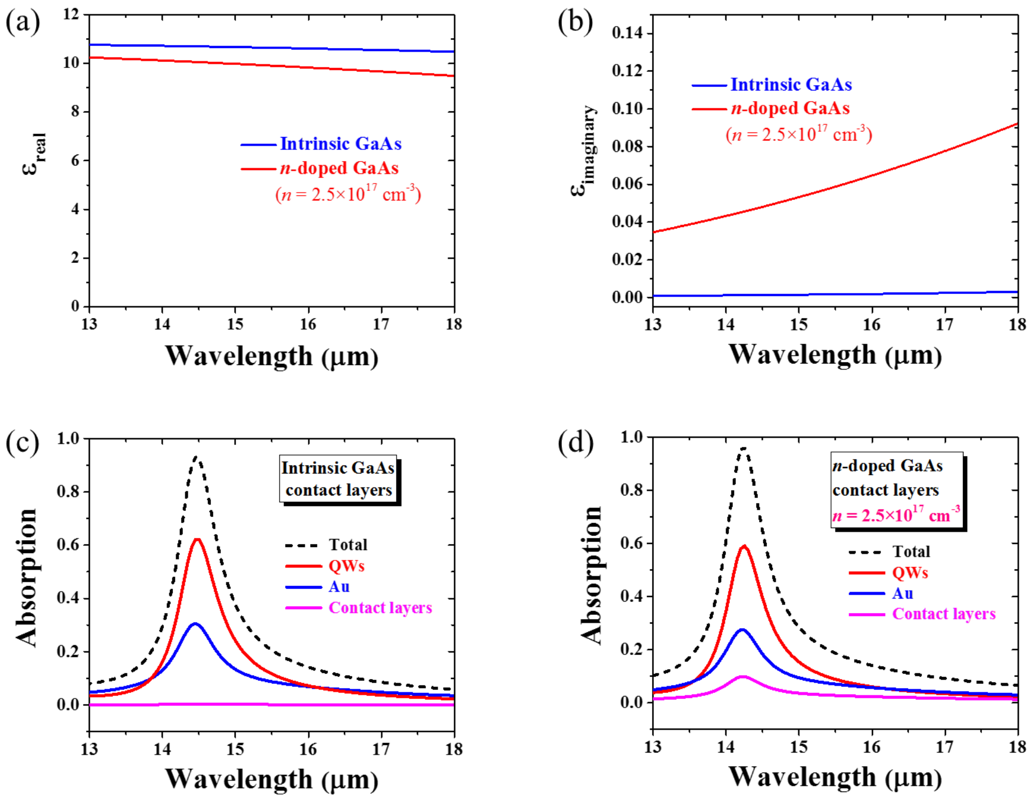

FIG. S1. Real (a) and imaginary (b) parts of the permittivities of the intrinsic (Blue line) and *n*-doped GaAs (Red line) with doping concentration of $n = 2.5\times10^{17}$ cm^{-3}. The absorptions in different layers under normal incidence of infrared radiation for the purposed QWIPs using intrinsic GaAs contact layers (c) and *n*-doped ($n = 2.5\times10^{17}$ cm^{-3}) contact layers (d), respectively.

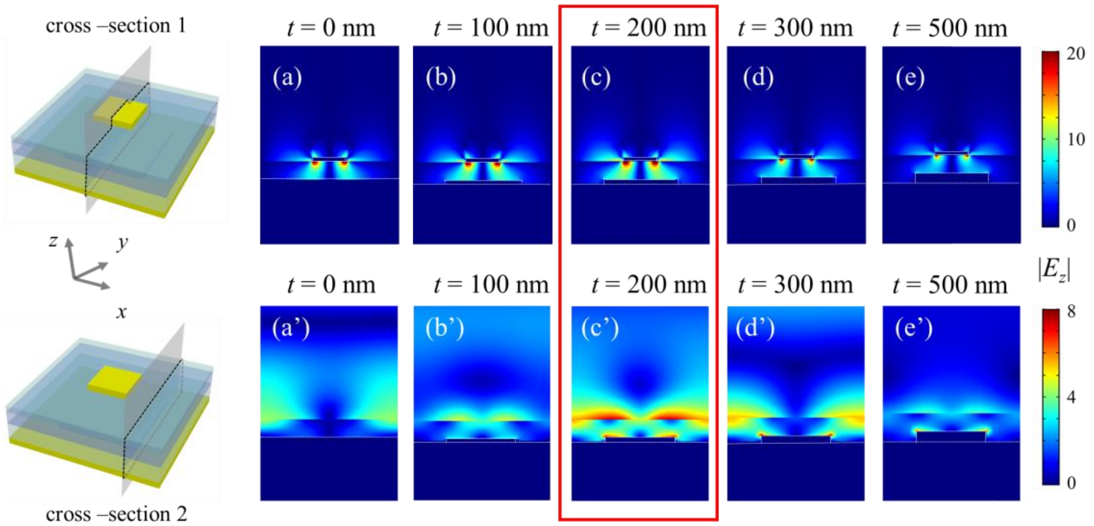

FIG. S2. For two typical cross-sections, distributions of time averaged $|E_z|$ for the absorption peak according to Fig. 5(b) with different t. The shapes of the gold frameworks are sketched in the figures with white dashed lines. To make it clear, an additional field distribution for the metallic optical incoupler with t = 500 nm is shown as (e) and (e'). The cross-section 1 is along the center of the top micropatch in the z-y plane (upper panel). The cross-section 2 is along the edge of the corrugated gold bulge in the z-y plane (lower panel). The other geometric parameters of the metallic optical incoupler are set as a = 1.6 µm, b = 3.5 µm. The field distributions outlined by the red box represent the case of the most optimized absorption using the purposed metallic optical incoupler for QWIPs with t = 200 nm.
